# Lipedema and obesity: biological interactions, diagnostic challenges, and principles of interdisciplinary management – a narrative review

**DOI:** 10.1080/21623945.2026.2719267

**Published:** 2026-08-20

**Authors:** Deborah Scherkamp, Victoria Wachenfeld-Teschner, Larissa Rabe, Fábio dos Santos Adrego, Gerrit Freund, Andreas Kroh, Andrea Schenk, Justus P. Beier, Anja M. Boos

**Affiliations:** a Department of Plastic Surgery, Hand Surgery and Burn Center, RWTH Aachen University Hospital, Aachen, Germany; b Department of Plastic Surgery, Hand Surgery, Burn Unit, University Hospital of Schleswig-Holstein, Lübeck, Germany; c Department of General, Visceral and Transplantation Surgery, RWTH Aachen University Hospital, Aachen, Germany; d Department of General, Visceral, Thoracic and Transplant Surgery, University Hospital of Schleswig-Holstein, Lübeck, Germany

**Keywords:** Lipedema, obesity, lymphoedema, weight management, bariatric surgery

## Abstract

Lipedema and obesity are chronic, frequently progressive disorders that can present with increased adipose-tissue volume, functional impairment and reduced quality of life. Although often approached as differential diagnoses, they frequently coexist and may aggravate one another. This narrative review addresses how lipedema and obesity interact biologically and clinically and what evidence supports a staged interdisciplinary approach to diagnosis and management. Obesity is increasingly conceptualized as an adiposity-based chronic disease characterized by excess adipose, abnormal adipose-tissue distribution or dysfunction, and associated medical or functional impairment. Obesity can impair lymphatic morphology and function, whereas lymphatic dysfunction may promote subcutaneous adipose-tissue expansion and fibrosis. Literature was identified through iterative PubMed searches and focused supplementary searches in Embase and the Cochrane Library. Publications were selected purposively for their relevance to predefined clinical and mechanistic domains; no systematic screening or formal risk-of-bias assessment was performed. This review synthesizes clinical overlap, biological evidence, diagnostic implications, and staged management for predominantly adult women with suspected or confirmed lipedema, including those with coexisting overweight or obesity. The proposed framework is intended to support clinical reasoning rather than serve as a formal guideline.

## Introduction

Lipedema and obesity are chronic, often progressive disorders that may both present with increased adipose-tissue volume, body circumference, functional impairment, and psychological burden. Their differentiation is complicated by phenotypic overlap, frequent coexistence, heterogeneous diagnostic criteria, and incomplete knowledge of the biological links among adipose tissue, inflammation, vascular permeability, and lymphatic dysfunction. Obesity has traditionally been classified using BMI thresholds, including BMI ≥ 30 kg/m^2^. However, recent EASO and Lancet frameworks conceptualize obesity in terms of excess adiposity, adipose-tissue distribution and dysfunction, and associated medical, functional, or psychological impairment; BMI is retained primarily as a screening and risk-stratification measure and should be supplemented by measures such as waist circumference, waist-to-height ratio, or body-composition assessment [1,2]. Lipedema is diagnosed clinically by symmetric, disproportional adipose-tissue accumulation in the extremities together with symptoms such as pain or pressure, tension, and heaviness. The point of departure for this review is that lipedema and obesity should be assessed as potentially coexisting and mutually modifying conditions rather than as mutually exclusive diagnoses. Th guiding question is: How do lipedema and obesity interact biologically and clinically, and what evidence supports a staged interdisciplinary approach to diagnosis and management. This review focuses primarily on adult women with suspected or confirmed lipedema, including those with coexisting overweight or obesity, and is intended for clinicians involved in diagnosis, conservative care, obesity treatment, and surgical management. It first addresses clinical definitions, differential diagnosis, and comorbidity; it then evaluates biological interactions using explicit levels of certainty; finally, it derives implications for staged, individualized management. This narrative synthesis is intended to support clinical reasoning and research prioritization, not to replace formal diagnostic or treatment guidelines.

## Review design and literature identification

This article was designed as a narrative review because its purpose is to integrate heterogeneous experimental, translational, epidemiological, and clinical evidence rather than to estimate a pooled treatment effect or exhaustively map a single, narrowly defined evidence base.

Literature was identified through iterative searches in PubMed, Embase and the Cochrane Library during manuscript development. Search concepts combined ‘lipedema’ or ‘lipoedema’ with ‘obesity’, ‘overweight’, ‘adipose tissue’, ‘lymphatic dysfunction’, ‘lymphedema’, ‘inflammation’, ‘vascular dysfunction’, ‘fibrosis’, ‘diet’, ‘supplementation’, ‘weight management’, ‘bariatric surgery’, and ‘liposuction’.

Publications were selected purposively according to their direct relevance to four predefined domains: definitions and differential diagnosis of lipedema and obesity; epidemiological and clinical overlap; adipose-tissue, immune, vascular, and lymphatic mechanisms; and implications for conservative, pharmacological, bariatric, liposuction, and post-bariatric management. Studies addressing lipedema or obesity without a discernible contribution to their biological or clinical interaction were not prioritized. Preference was given to clinical guidelines and consensus statements, systematic reviews, human cohort studies, and mechanistic studies directly examining lipedema or obesity-associated lymphatic dysfunction.

No prospectively registered protocol, formal duplicate screening procedure, standardized risk-of-bias assessment, or meta-analysis was used. Evidence was synthesized qualitatively with attention to study design, directness, consistency, and clinical relevance, and observed associations were distinguished from proposed and more firmly supported mechanisms. The preparation and reporting of the review were informed by the SANRA quality domains [3]. Table 1 summarizes representative studies and a qualitative interpretation of evidentiary confidence; these labels are narrative judgements.

**Table 1.** Representative studies underpinning major claims and their evidentiary limitations.

Wolf et al. (12) – small cross-sectional case-control laboratory study with in vitro analyses; up to *n* = 20 lipedema and *n* = 10 BMI-matched controls	Surgical adipose-tissue and serum samples	Reported cytokine differences, larger adipocytes, altered stromal-vascular-fraction metabolism, and no major lipid-composition difference.	Small surgery-selected sample; cross-sectional design; assay-specific sample subsets; multiple experimental endpoints; no clinical outcomes.	Hypothesis-generating mechanistic evidence. Low confidence for causality or clinical actionability.
Suga et al. [46] - histopathological case study; *n* = 1	One patient with severe lipedema; affected and non-affected tissue compared within the patient	Observed adipocyte hypertrophy, adipocyte death, and concurrent regenerative features.	Single case; no independent control cohort; cannot assess variability or temporality.	Illustrative mechanistic observation. Very low confidence and not generalizable.
Felmerer et al. [50] - cross-sectional matched case-control study; *n* = 11 lipedema and *n* = 10 controls	Anatomically and BMI-matched tissue and serum samples	Reported higher systemic VEGF-C and macrophage infiltration without clear changes in lymphatic-vessel morphology.	Small surgery-selected cohort; cross-sectional measurements; no temporal or functional clinical validation.	Hypothesis-generating association. Low confidence for causal direction.
Kruppa et al. [47] - cross-sectional tissue study; *n* = 32 lipedema and *n* = 14 controls, with smaller assay-specific subsets	Stages I-III compared with age- and BMI-matched controls	Reported stage-associated thigh adipocyte hypertrophy, fibrosis, inflammatory markers, and macrophage patterns.	Small stage subgroups; multiple comparisons; possible stage-BMI confounding; no longitudinal follow-up.	Supports a tissue phenotype and stage association. Low confidence that differences represent progression or causality.
Chachaj et al. [56] - cross-sectional lymphoscintigraphic comparison; *n* = 51 lipedema and *n* = 31 overweight or obesity	Women without clinical lymphoedema	Found predominantly minor or moderate lymphoscintigraphic alterations in both groups and limited ability to distinguish lipedema from overweight or obesity.	No healthy-weight control; cross-sectional design; diagnostic heterogeneity; imaging abnormalities were not equivalent to clinical lymphoedema.	Low-confidence evidence for subclinical lymphatic alterations; not evidence of diagnostic specificity or causal direction.
Baumgartner et al. [13] - single-centre uncontrolled long-term follow-up; *n* = 60	Patients assessed approximately 12 years after liposuction	Reported sustained patient-rated symptom improvement and reduced use of conservative therapy.	No concurrent control; questionnaire and recall bias; attrition and selection bias; no blinding or standardized comparative outcome assessment.	Suggests durability in selected patients. Low confidence for comparative treatment effectiveness.
Fink et al. [72] - retrospective cohort; *n* = 31 lipedema, comparator *n* = 14	Patients undergoing sleeve gastrectomy or Roux-en-Y gastric bypass	Thigh volume decreased after bariatric surgery and was broadly comparable with the control trajectory.	Small retrospective sample; no randomization; possible diagnostic misclassification; focus on volume rather than pain, function, or quality of life.	Low-confidence lipedema-specific evidence. Supports volume reduction but not symptom resolution or comparative effectiveness.
de Camargo et al. [77] - systematic review available as a conference abstract only	Diet and supplementation as conservative treatment for lipedema	Indicates an emerging literature on dietary and supplement interventions.	Full search strategy, included-study characteristics, risk-of-bias assessment, and effect estimates cannot be appraised from the abstract.	Very low confidence for clinical recommendations; hypothesis-generating only.
Busetto et al. and Rubino et al. [75,76] - expert frameworks / consensus statements; no patient sample	Adults with obesity or excess adiposity	Recommend diagnosis and staging based on adiposity distribution, tissue or organ dysfunction, complications, and function rather than BMI alone.	Consensus-based frameworks, not trials of diagnostic thresholds; implementation and outcome validation are evolving.	Clinically influential definitions. They do not provide evidence of treatment effectiveness.
Faerber et al. [79] - S2k multidisciplinary consensus guideline; no patient sample	Patients with suspected or diagnosed lipedema; clinical guideline context	Provides consensus recommendations for clinical diagnosis, pain-oriented management, obesity assessment, conservative care, and selection for liposuction.	Consensus methodology does not establish diagnostic accuracy or comparative treatment effectiveness; some recommendations rely on limited direct evidence.	Clinically actionable consensus guidance. Certainty varies by recommendation and should not be equated with trial evidence.
Blome et al. [81] - instrument development and validation; cross-sectional *n* = 301 and longitudinal randomized study *n* = 82	Patients with lymphoedema, lipedema, or lipolymphedema receiving oedema treatment	Developed the PBI-L and reported internal consistency, construct validity, and responsiveness for patient-relevant treatment benefit.	Mixed diagnostic populations; not designed to determine treatment effectiveness or a lipedema-specific minimal important difference.	Moderate evidence that PBI-L is a suitable patient-reported benefit instrument; not an efficacy estimate.
Eason et al. [82] - systematic review; 20 studies using 13 assessment tools	Studies using physical or imaging measures for lipedema diagnosis or treatment outcomes	Identified substantial heterogeneity in tools, protocols, anatomical sites, and outcome analysis.	Small and heterogeneous primary studies with limited clinimetric reporting; no single tool could be recommended as a gold standard.	Moderate confidence in the conclusion that measurement standardization is lacking; low confidence for choosing one superior tool.
Mortada et al. [18] – systematic review and meta-analysis; 20 predominantly observational studies, 9 included in meta-analysis	Patients with lipedema undergoing different liposuction modalities	Reported improvements in patient-reported symptoms and quality of life, with serious complications infrequently reported.	No high-quality randomized comparative trials; heterogeneous techniques and outcome measures; meta-analysis inherits bias of uncontrolled studies.	Low-certainty evidence of benefit in selected patients; insufficient for a precise individual effect size or comparative effectiveness.

### Clinical definitions, diagnostic overlap, and comorbidity

#### Phenotypic overlap among disorders of subcutaneous adipose tissue

In addition to lipedema and obesity, several disorders are characterized by abnormal subcutaneous adipose-tissue distribution [4]. While both generalized and partial lipodystrophy are associated with a reduction in subcutaneous adipose tissue, which can lead to severe metabolic disturbances, including consequences of leptin deficiency, most of these diseases are associated with an increase in subcutaneous adipose tissue [5,6]. Whereas lipomas are localized, encapsulated deposits of adipose tissue, which may occur as solitary or multiple lesions, lipomatosis, which can occur in isolation or be part of a syndrome, is characterized by a diffuse but regionally confined increase in adipose tissue in certain areas of the body. Distinct clinical subtypes of lipomatosis can be differentiated. Dercum’s disease, also known as adiposis dolorosa, mostly affects women and is associated with the formation of painful nodules. Roch-Leri mesosomatous lipomatosis predominantly affects men and is generally painless [7–9]. Other disorders involving increased subcutaneous adipose tissue, such as obesity, lipedema, and lymphoedema, are associated with a widespread increase in adipose tissue in the affected regions. In both secondary and primary lymphoedema, there is damage to the lymphatic system. This dysfunction initially causes oedema through the accumulation of interstitial fluid in the affected regions. The high protein content of lymph fluid causes an inflammatory response with increased fibroblastic activity. This, along with the deposition of lipids in the lymph, leads to tissue changes that are typical of the later stages of lymphoedema and are characterized by a lack of compressibility due to fibrotic remodelling and adipose tissue hypertrophy. This results in an adipose tissue distribution disorder, which often only affects individual extremities and can therefore be distinguished from both obesity and lipedema [10,11]. Although several of these conditions can be differentiated clinically, overlapping phenotypes, uncertain mechanisms, and frequent coexistence continue to complicate diagnosis and treatment.

#### Pathophysiological uncertainty complicates the diagnosis and treatment of lipedema

Lipedema is a chronic, progressive disease characterized by a disproportionate, symmetric increase in adipose tissue in the extremities. It leads to limitations in daily and occupational functioning and is associated with a substantial reduction in quality of life. Lipedema mainly affects women; in men, the disease has been reported only rarely, typically in association with disturbances of sex- or growth-hormone regulation [12]. Published prevalence estimates vary widely, and the frequently cited estimate of approximately 10% of women should be interpreted cautiously. Robust population-based studies are scarce, diagnostic criteria and case definitions vary, and many estimates derive from selected clinical populations, creating substantial risks of referral and ascertainment bias [13].

Although first described by Allen and Hines in 1940, research and public awareness have increased mainly in recent years [14]. However, despite the increased interest, the underlying pathogenesis of lipedema remains incompletely understood, complicating diagnosis and treatment. The treatment of lipedema includes conservative and surgical methods, selected according to disease severity and patient impairment. Treatment primarily aims to relieve symptoms; current approaches do not consistently reduce volume and are not curative. Conservative management, particularly complex physical decongestive therapy, forms the basis of treatment. This includes manual lymphatic drainage, compression therapy with compression garments, and exercise therapy. Psychosocial support, patient education, and access to self-help groups may facilitate coping and self-management. Liposuction may be considered when symptoms persist despite conservative therapy. Although volume reduction is not achieved in every patient, studies report symptom improvement and reduced requirements for conservative therapy [15,16]. The direct evidence for liposuction consists mainly of uncontrolled cohorts and patient-reported follow-up rather than randomized comparisons. The 12-year study by Baumgartner et al. was a single-centre follow-up of 60 patients without a concurrent control group; it therefore suggests possible durability in selected patients but does not establish comparative effectiveness [16]. Current S2k guidance recommends that candidacy for liposuction be based on documented treatment-resistant pain and/or clinically relevant functional, dermatological, or orthopaedic complications rather than morphological stage alone. Coexisting obesity should be treated as a separate disease; a BMI above 40 kg/m^2^ together with a waist-to-height ratio above 0.55 is described as a critical indication constellation, not as an absolute contraindication, and clinically relevant oedema should be treated before surgery [17]. A recent systematic review and meta-analysis reported improvements in symptoms and quality of life after liposuction, but the included evidence was predominantly observational and did not establish comparative effectiveness or a reliable, expected effect size for an individual patient [18].

Diagnosis and staging of lipedema are based primarily on clinical assessments, including the medical history, physical examination, and the exclusion of alternative diagnoses. A positive family history and the onset during puberty, pregnancy, or menopause may support the diagnosis. Physical examination determines the distribution type: type I affects the buttocks, type II the thighs, type V the lower legs, and type III the entire legs. In type IV, there is a symmetrical increase of adipose tissue on the upper arms, usually in combination with type II or III. Severity is traditionally graded by palpation and skin morphology. Stage I is characterized by small palpable nodules and a smooth skin surface, stage II by larger, firmer nodules and an uneven surface; and stage III by pronounced tissue irregularities and overhanging fat lobes. Additionalclinical features include easy bruising, telangiectasia, pain on pressure or touch, and sensations of tension and heaviness in the affected regions. Differentiation from other causes of limb enlargement remains essential. The principal clinical boundaries among lipedema, obesity, primary or secondary lymphoedema, Dercum disease, lipodystrophy, and lipomatosis are summarized in Table 2. The listed features are orientation points rather than individually diagnostic findings, and coexistence must be considered.

**Table 2.** Practical differential diagnosis of disorders affecting subcutaneous adipose tissue and limb volume.

Condition	Distribution and symptoms	Oedema, Stemmer sign, and weight-loss response	Diagnostic role and clinical clues
Lipedema	**Distribution**: Bilateral, symmetric, disproportionate subcutaneous adipose tissue of legs and sometimes arms; hands and feet usually spared; ankle or wrist cuff may be present. **Pain**: Pressure or touch pain, spontaneous pain, heaviness, tension, and easy bruising are characteristic but variable.	**Oedema**: Pitting oedema is not required. Orthostatic swelling or coexisting secondary lymphoedema may occur. **Stemmer sign**: Usually negative; a positive sign should prompt assessment for coexisting lymphoedema. **Weight loss**: General and limb volume may decrease when obesity coexists, but disproportion and symptoms may persist; response is variable.	**Diagnosis/imaging**: Clinical diagnosis; no confirmatory laboratory or imaging test. Imaging is reserved for differential questions or suspected lymphatic or venous disease. **Typical clues**: Obesity, reduced mobility, chronic pain, psychosocial distress; onset around hormonal transitions and family history may support but do not prove diagnosis.
Obesity	**Distribution**: Generalized, central, or regional excess adiposity; limb enlargement is usually proportionate to overall adiposity, although distribution varies. **Pain**: Adipose tissue is not typically painful on pressure; pain more often reflects musculoskeletal or other comorbidity.	**Oedema**: Not intrinsic to obesity, but venous disease, cardiac or renal disease, medication effects, or obesity-associated lymphoedema may cause oedema. **Stemmer sign**: Negative unless lymphoedema coexists. **Weight loss**: Effective obesity treatment reduces total adiposity; regional response and symptom response vary.	**Diagnosis/imaging**: BMI is a screening measure. Waist measures, body composition where available, and adiposity-related organ or functional dysfunction improve characterization. **Typical clues**: Metabolic, cardiovascular, hepatic, respiratory, and osteoarticular complications; psychological burden and stigma.
Primary or secondary lymphedema	**Distribution**: Often distal and may include feet or hands; frequently asymmetric, although primary disease can be bilateral. **Pain**: Heaviness and tightness are common; pain is variable and is not the defining feature.	**Oedema**: Pitting may occur early; later fibrosis, skin thickening, and reduced compressibility can develop. **Stemmer sign**: Often positive in established disease but may be negative in early lymphoedema. **Weight loss**: Fluid and fibrotic tissue do not resolve through weight loss alone; weight reduction may reduce mechanical and inflammatory load when obesity coexists.	**Diagnosis/imaging**: Clinical diagnosis supported when needed by venous ultrasound, lymphoscintigraphy, indocyanine-green lymphography, or MR lymphangiography. **Typical clues**: Cellulitis and skin changes; primary onset patterns or secondary causes such as cancer treatment, infection, trauma, or severe obesity.
Dercum disease	**Distribution**: Generalized or regional painful adipose tissue with multiple painful lipomas or nodules, often involving trunk and proximal limbs. **Pain**: Chronic pain and tenderness are defining clinical features.	**Oedema**: Oedema is not a defining feature. **Stemmer sign**: Usually negative unless lymphoedema coexists. **Weight loss**: Weight loss may reduce general adiposity but does not reliably remove painful nodules or pain.	**Diagnosis/imaging**: Clinical diagnosis; ultrasound or MRI can characterize focal masses and exclude alternatives. **Typical clues**: Obesity is common; fatigue, sleep disturbance, and psychological symptoms may coexist, but are not specific.
Lipodystrophy	**Distribution**: Generalized or partial loss of subcutaneous adipose tissue, sometimes with regional fat accumulation and prominent muscular appearance. **Pain**: Pain is not a defining feature.	**Oedema**: Oedema is not typical. **Stemmer sign**: Negative unless a separate lymphatic disorder exists. **Weight loss**: Not primarily corrected by conventional weight loss; management targets metabolic complications and the specific syndrome.	**Diagnosis/imaging**: Body-composition assessment, metabolic laboratory evaluation, and selected genetic testing may support diagnosis. **Typical clues**: Severe insulin resistance, diabetes, hypertriglyceridaemia, fatty liver, and low leptin in selected forms.
Lipomatosis	**Distribution**: Multiple or diffuse regionally confined adipose masses; pattern depends on subtype and may involve neck, trunk, or limbs. **Pain**: Usually painless, although compression symptoms or painful adipose disorders may occur.	**Oedema**: Oedema is not typical. **Stemmer sign**: Negative unless lymphoedema coexists. **Weight loss**: Established masses often persist despite weight loss.	**Diagnosis/imaging**: Ultrasound or MRI can define the number, distribution, and extent of masses. **Typical clues**: Family history, alcohol exposure, neuropathy, or syndromic features may be present depending on subtype.

#### Reported overweight and obesity in lipedema cohorts

Beyond limb enlargement, reduced mobility, heaviness, tension, and pain on pressure or touch, patients with lipedema report numerous comorbidities. In a study involving 245 patients with lipedema that assessed comorbidities, sociodemographic characteristics, disease features, symptom severity, and psychological effects, 93.5% reported at least one comorbidity. These included allergies, hypertension, asthma, hypothyroidism, migraine, fibromyalgia, gastrointestinal disorders, eating disorders, rheumatic disorders, and type 2 diabetes; most participants (62.9%) were aged 40–59 years [19]. Czerwińska et al. analysed four additional studies and concluded that only approximately 25% of patients had no comorbidity other than lipedema [20]. Across these studies, overweight or obesity was the most frequently reported comorbidity. Falck et al. reported overweight or obesity in 30.2% of the lipedema patients, with obesity more frequent at higher lipedema stages whereby [19]. Ghods et al. reported obesity in37.6% of 147 patients and in 87.1% of patients with stage III lipedema [21]. Bertsch et al. reported that 97% of 2,344 women diagnosed with lipedema at the authors’ lymphology clinic were overweight (9%) or obese (88%) [22]. These figures show that excess weight is common in speciality cohorts but should not be interpreted as population prevalence. Most studies are clinic-based, use heterogeneous diagnostic criteria, and preferentially include patients with more severe symptoms; referral, selection, and diagnostic-verification bias therefore limit generalizability. Falck et al. used a cross-sectional survey, whereas Ghods et al. conducted a retrospective speciality-centre analysis; neither design can establish that obesity causes lipedema progression or provide an unbiased population estimate [19,21].

### Biological interactions and levels of mechanistic evidence

#### Obesity as an adiposity-based chronic disease

**Figure 1.**
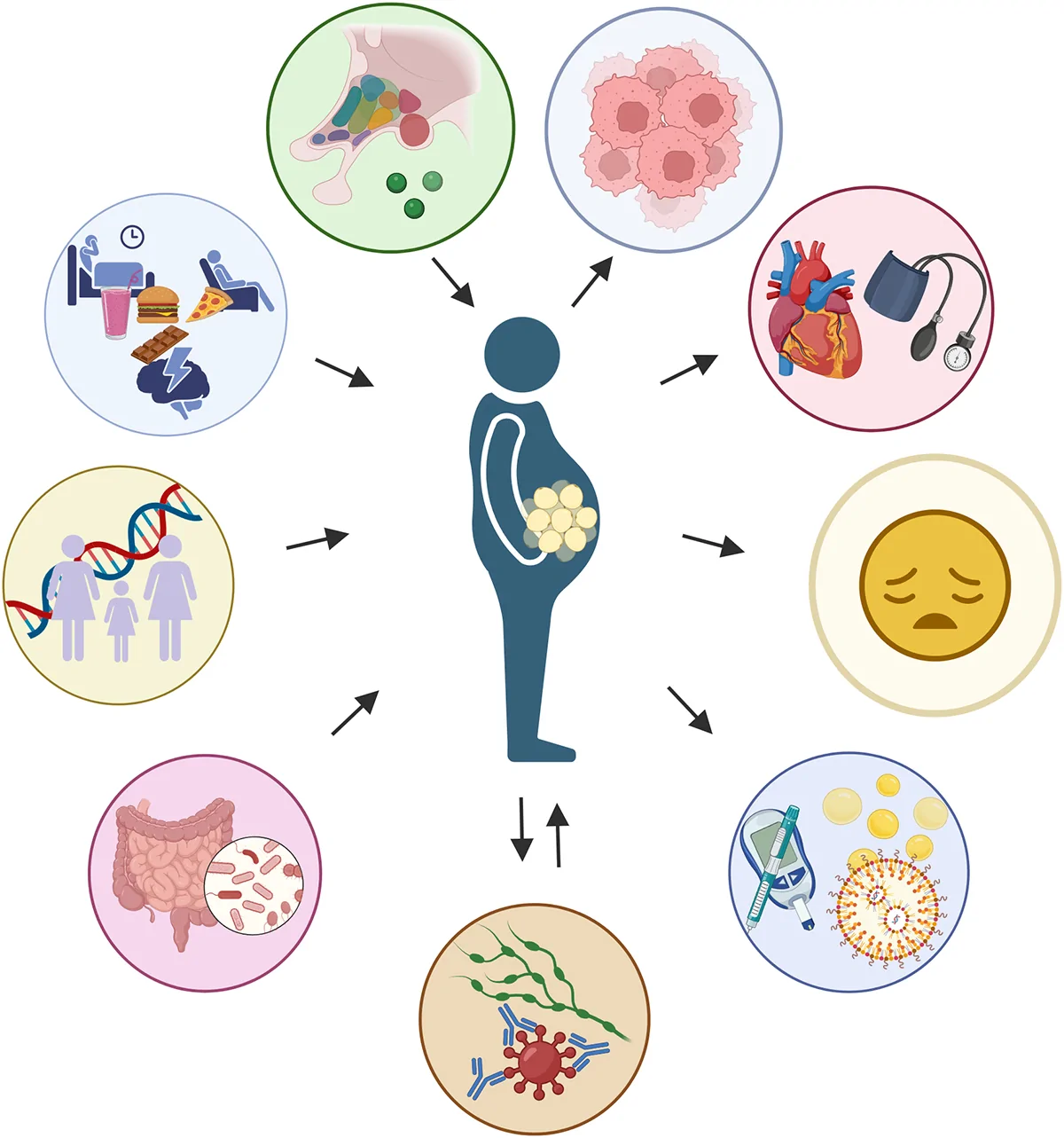
Factors involved in the development and systemic effects of obesity. Obesity is influenced by lifestyle factors including diet, physical activity, sleep deprivation, and chronic stress, as well as neural circuits, inflammation, the gut microbiome, family history, genetics and epigenetics. It is associated with inflammation, metabolic and cardiovascular diseases, cancer risk, and reduced quality of life. Evidence category: established general-obesity associations and risk relationships; the schematic is not lipedema-specific and does not imply that every pathway occurs in every patient.

Population estimates have traditionally been reported using BMI-based categories. According to World Health Organization data for 2016, more than 1.6 billion adults (39%) were classified as overweight (body mass index, ≥25 kg/m^2^), including 650 million (13%) classified as obese (BMI ≥ 30 kg/m^2^), and the prevalence of obesity had nearly tripled over four decades [23]. These figures describe BMI categories and should not be equated with an individual diagnosis of adiposity-related disease. Recent EASO and Lancet frameworks conceptualize obesity as an adiposity-based chronic condition and recommend assessment of excess adiposity, fat distribution, adipose-tissue dysfunction, and associated medical, functional, or psychological impairment [1,2]. BMI remains useful for screening, epidemiology, and some treatment thresholds, but it does not directly measure fat mass, regional distribution, or adipose-tissue function. This limitation is particularly relevant in lipedema, in which disproportionate peripheral subcutaneous adipose tissue may increase BMI without reflecting the same risk profile as visceral adiposity. Obesity involves a chronic imbalance between energy intake and expenditure [24]. An increase in energy-dense food (sugar and fat) and physical inactivity were historically considered the principal drivers of the increasing prevalence of obesity. Obesity, however, is a multifactorial disease rather than a simple consequence of excess caloric intake. According to Ho et al, obesity is a polygenic disease shaped by interacting factors, including neural circuits, hormones, nutrient absorption, lipid storage, lipid metabolism, and inflammation [25]. Evidence also indicates that family history, lifestyle, and psychological factors influence body weight [26]. The consumption of energy-rich foods and reduced physical activity, as well as other lifestyle factors such as sleep deprivation or chronic stress, also contribute to the development of obesity [27,28]. In addition, genes that increase the risk of obesity have been identified, but collectively explain only a limited proportion of obesity risk [29]. Epigenetic changes also contribute to the development of obesity, for example, via DNA methylation of the leptin profile [30]. It has been shown that both undernutrition and overnutrition of the mother during the foetal period and breastfeeding increase the risk of obesity and metabolic diseases [31]. Hypothalamic pathways, among others, play a decisive role in mediating these effects. Metabolic signals such as nutrients and hormones as well as external signals, such as food availability, are processed in the brain and serve to maintain energy homoeostasis [31]. Studies also suggest that alterations in the gut microbiome influence the development of obesity, although the exact mechanism has not yet been clarified [26]. Obesity also induces significant physiological changes in the body and thus increases the risk of additional comorbidities (Figure 1). Adipose tissue is not merely an energy store and thermal insulator; it also has endocrine and immune functions [32]. In addition to adipocytes and preadipocytes, adipose tissue consists of fibroblasts and immune cells and undergoes obesity-associated changes in cellular composition and function [33]. Obesity reduces the homoeostatic function of adipose tissue, thereby leading to numerous metabolic disorders. For example, excess adipose tissue and hypertrophic adipocytes have a pro-inflammatory effect via the secretion of CRP, interleukins (IL1 β, IL6, IL 34) and tumour necrosis factor alpha (TNFα). Additionally, anti-inflammatory mediators, such as adiponectin, are reduced [34,35]. The resulting local and systemic inflammation leads to insulin resistance, thereby contributing to systemic metabolic dysfunction [32]. In addition to promoting insulin resistance and type 2 diabetes, obesity is associated with reduced quality of life, cardiovascular diseases, musculoskeletal disorders like osteoarthritis, cancer, hypertension, hypercholesterolemia, hyperlipidaemia, and stroke [25,36,37]. Accordingly, overweight and obesity are associated with increased mortality [38,39].

## Bidirectional interactions between adipose tissue and the lymphatic system

Overweight has been identified as a risk factor for the development of secondary lymphoedema, as shown by Helyer et al. and Werner et al. in studies with cancer patients undergoing lymph node dissection [40,41]. Potential mechanisms include obesity-associated changes in lymphatic-vessel morphology and size, reduced contractility, increased permeability, and decreased expression of regulators of lymphatic proliferation and function, such as VEGFR3 and PROX-1 [42]. Furthermore, Silha et al. reported increased secretion of VEGF-C by adipocytes during expansion, which in turn leads to lymphatic hyperplasia [16]. In addition to altering lymphatic vessels, obesity reduces lymph-node size, further impairing lymph transport [43]. The cancer-related studies are observational risk-factor analyses and support an association between higher body weight and postoperative lymphoedema. By contrast, the claims concerning vessel morphology, contractility, and molecular regulators arise largely from experimental or preclinical work; these evidence streams should not be interpreted as equivalent in clinical certainty.

**Figure 2.**
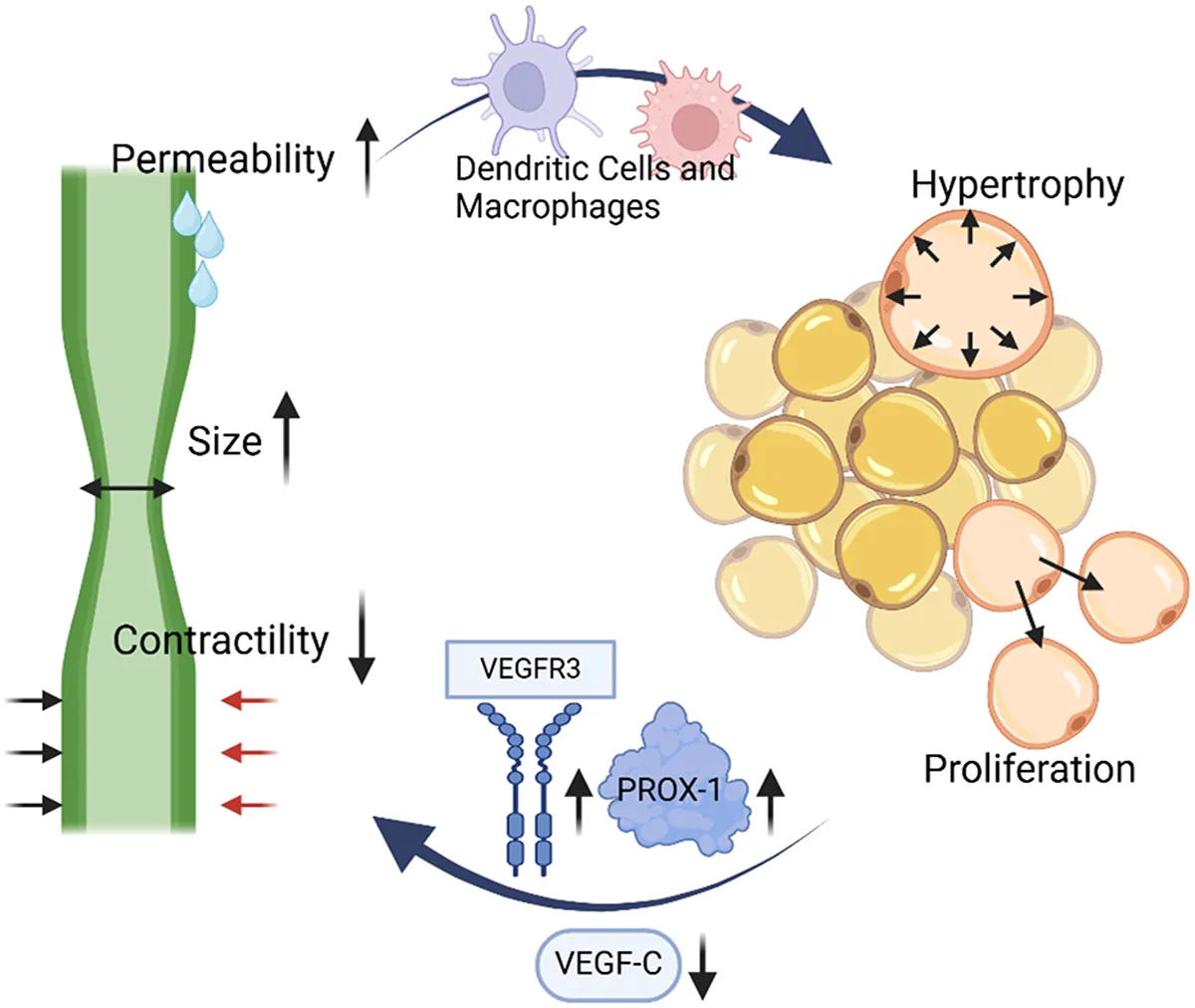
Bidirectional interactions between obesity and lymphatic dysfunction. Overweight and obesity may promote secondary lymphoedema by altering lymphatic vessel morphology and size, reducing contractility, increasing permeability, and decreasing regulators of lymphatic proliferation and function. Conversely, lymphoedema promotes macrophage and lymphocyte infiltration, adipocyte proliferation and hypertrophy, adipose-tissue accumulation and fibrosis. Evidence category: conceptual synthesis based on mixed human observational and experimental evidence. Arrows indicate proposed bidirectionality rather than an established causal sequence.

Conversely, lymphoedema is associated with the infiltration of macrophages and lymphocytes in the affected tissue, promoting proliferation and hypertrophy of adipocytes [44]. The lymphatic vasculature also regulates lipid absorption and inflammation [25]. Accordingly, impaired lymphatic function may promote adipose tissue accumulation, predominantly in the subcutaneous compartment, and fibrosis [45]. This observation is consistent with the adipogenesis caused by lymphatic fluid described by Schneider et al. and supports a mechanistic link between pathological changes in the lymphatic vessels, fluid accumulation in the tissue, and adipose tissue hypertrophy (15, Figure 2). Accordingly, the proposed bidirectional feedback loop is biologically plausible but has not been directly demonstrated in prospective human studies of lipedema.

## Biological findings and proposed mechanisms in lipedema

**Figure 3.**
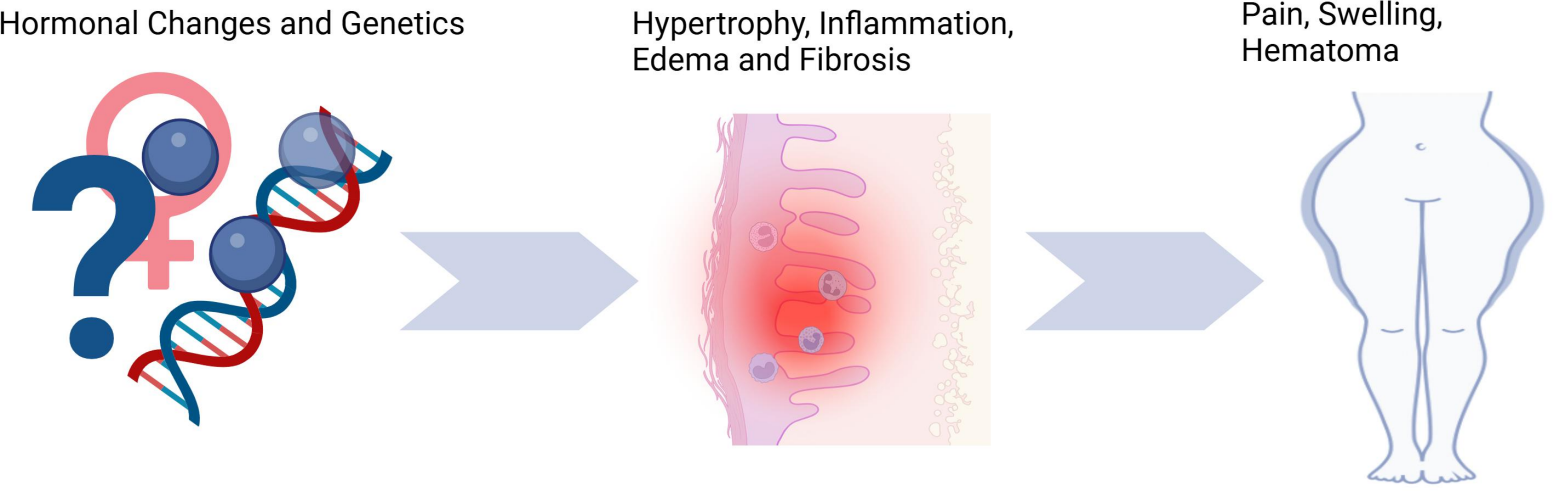
Conceptual model of proposed mechanisms and clinical manifestations of lipedema. Genetic susceptibility and hormonal transitions may contribute to regional adipose-tissue changes, inflammation, oedema, and fibrosis of the affected tissue, resulting in pain, swelling, and easy bruising. The depicted relationships are hypotheses based mainly on observational and experimental findings and should not be interpreted as established causal pathways. Evidence category: proposed mechanisms supported mainly by associative and hypothesis-generating evidence.

To avoid conflating association with causation, the biological evidence is organized here into five levels. Proposed initiating factors include genetic susceptibility and hormonal transitions; these are supported by familial clustering and temporal associations but are not established causes. Observed tissue-level findings include adipocyte hypertrophy in some cohorts, stromal-vascular changes, extracellular-matrix remodelling, fibrosis, immune-cell infiltration, and increased vascular permeability. Hypoxia, adipocyte necrosis, macrophage polarization, and lymphatic dysfunction are plausible amplification mechanisms, but their temporal sequence and causal relevance remain uncertain (Figure 3). The clinical phenotype (disproportionate painful limb adiposity, easy bruising, heaviness, and functional limitation) is well described, whereas the biological pathway that produces it is not. Obesity should therefore be considered a potential modifier that may increase adipose-tissue load, inflammation, mechanical impairment, and lymphatic stress rather than a proven initiating cause of lipedema. Overall, most mechanistic evidence is cross-sectional, derived from small cohorts, or extrapolated from experimental models.

Familial clustering is an observed association that suggests, but does not establish, a genetic contribution. Cellular alterations, dysregulated cytokine and growth-factor signalling, and hormonal changes have also been reported in lipedema [12,46]. Because onset is frequently reported during puberty, pregnancy, or menopause, sex hormones, including oestrogen and progesterone, have been proposed to contribute through altered receptor distribution; this temporal association does not demonstrate causality [12,47,48]. Transcriptomic analysis of lipedema-affected adipose tissue by Ishaq et al. identified differential expression of 4,391 genes, including genes associated with adipose-tissue proliferation, fibrosis, and inflammation [49]. These data identify candidate pathways but do not establish which changes are primary, secondary, or clinically causal. The transcriptomic study by Ishaq et al. was an exploratory mechanistic analysis; its pathway findings require independent replication and clinical validation before they can be considered biomarkers or therapeutic targets.

## Observed adipose-tissue changes: adipocyte hypertrophy, inflammation, and fibrosis

At the cellular level, adipocyte hypertrophy and expansion of the stromal vascular fraction (SVF) have been reported in small observational studies [15,50,51]. Findings are not uniform: some authors found no difference in the adipocyte size, whereas others, including Kruppa et al., reported stage dependent adipocyte hypertrophy [15,48,51,52]. Lipid composition in serum and lipoaspirate did not differ between lipedema and control samples in one study [15]. Increased cell numbers and mitochondrial activity have been reported in the SVF of patients with lipedema [15,50]. Pringlinger et al. found similar adipose-derived stromal-cell morphology in lipedema and control tissue but reduced adipogenic differentiation potential in lipedema-derived cells [50]. In contrast, Suga et al. reported increased CD34 and Ki67 expression and proposed that enhanced stromal-cell proliferation could promote adipogenesis, inadequate perfusion, hypoxia, and necrosis due to lack of sufficient blood supply [51]. Hypertrophy-induced hypoxia and subsequent necrosis are therefore proposed amplification mechanisms rather than established initiating events [53]. Extracellular-matrix remodelling, including increased sodium concentrations, collagen deposition, and glycocalyx alterations, has also been observed [54]. These findings support tissue remodelling in lipedema but do not establish a single causal sequence. The Suga et al. report was based on affected and unaffected tissue from a single patient and is illustrative rather than generalizable. The larger study by Kruppa et al. was cross-sectional, with small stage-specific subgroups, and therefore cannot show that between-stage differences represent longitudinal disease progression [51,52].

Although systemic inflammatory markers, including C-reactive protein and leukocyte counts, were not elevated, Felmerer et al. reported increased infiltration of CD45-positive immune cells lipedema tissue. This is an observed local association and does not determine whether inflammation precedes or follows adipose-tissue expansion and injury. T-cell number were unchanged in the study by Felmerer at al., whereas macrophage infiltration, particularly M2 macrophages, was increased [55]. Wolf et al. likewise reported more M2 macrophages and reduced lipid formation after pharmacologically inducing an M2-to-M1 shift in an experimental model [15]. Strohmeier et al. also observed increased CD11c positive cells, including macrophages and dendritic cells, and perivascular macrophage accumulation [48]. Other studies have described crown-like macrophage structures surrounding necrotic adipocytes [50,51,56]. Collectively, these observations support local immune involvement, but macrophage accumulation may be a cause, an amplifier, or a consequence of tissue injury.

## Observed vascular and lymphatic alterations in lipedema

**Figure 4.**
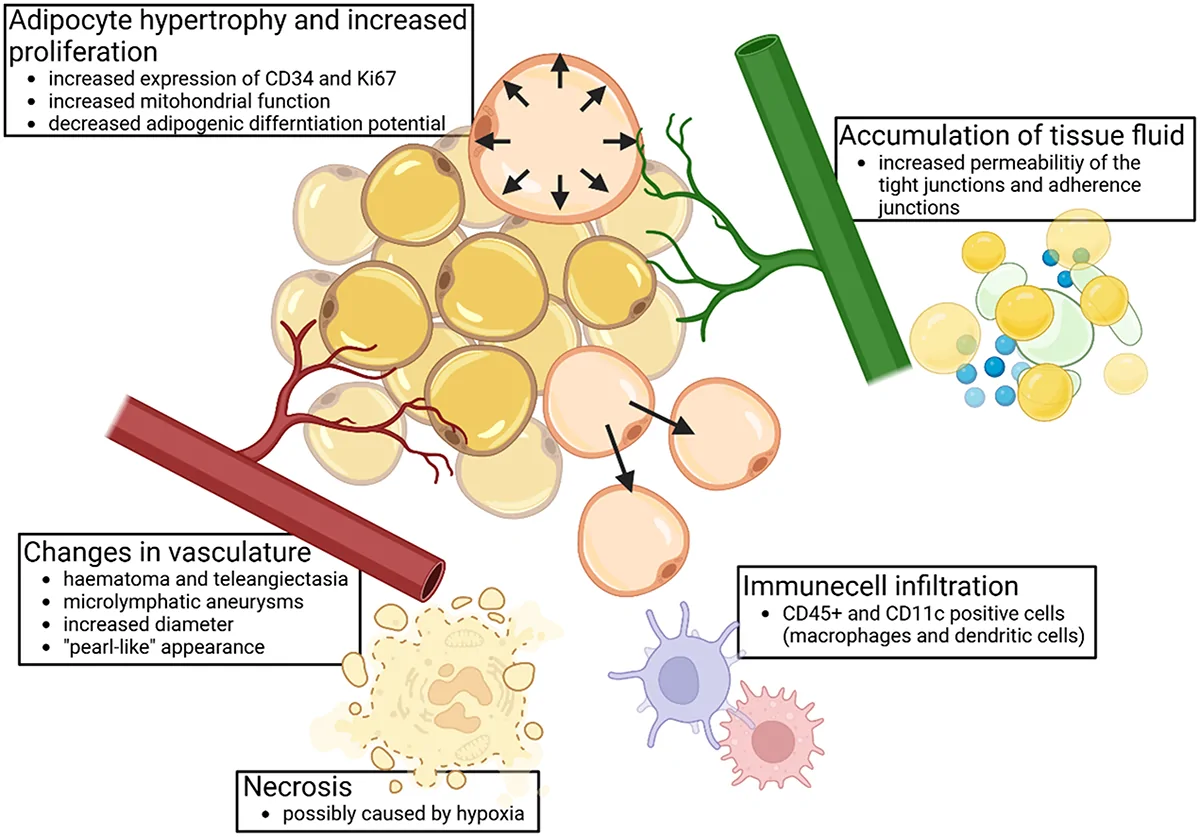
Observed findings and proposed amplification mechanisms in lipedema. Increased vascular permeability and interstitial-fluid accumulation have been reported. Adipocyte hypertrophy and proliferation may impair perfusion and promote hypoxia and necrosis, while CD45-positive and CD11c-positive immune cells may accumulate in response to tissue injury. The temporal order and causal direction of these processes remain uncertain. Evidence category: observed associations combined with proposed amplification mechanisms; causal order remains uncertain.

Clinical observations of telangiectasias and easy bruising in lipedema-affected regions suggests vascular involvement (Figure 4). Földi and Földi described microangiopathy as an early histological correlate in lipedema tissue [14]. However, vascular morphology has not been consistent across studies, some authors found no clear changes, whereas Al-Ghadban et al. reported increased vessel number and, in some samples, diameter when comparing non-obese patients with and without lipedema [55,56]. These cross-sectional findings establish association, not temporal or causal direction. Fluorescence imaging has shown microlymphatic aneurysms in the lower extremities of patients with lipedema [57], and MRI has demonstrated enlarged lymphatic vessels with a pearl-like appearance [58]. Lymphoscintigraphy and lymphangiography have reported delayed lymphatic drainage [59,60]. In contrast, immunohistochemical analyses using podoplanin, LYVE-1, CD31, and PROX-1 have not consistently demonstrated increased lymphatic-vessel number or diameter [48,55]. Increased permeability of endothelial tight and adherens junctions has been observed in lipedema tissue and induced in non-lipedema cells by exposure to lipedema-conditioned medium [55]. Together, these observations support vascular and lymphatic involvement, but inconsistent findings and cross-sectional designs preclude an established causal sequence.

## Reported lymphatic dysfunction in obesity and lipedema

Given bidirectional effects of obesity on lymphatic function and of lymphatic fluid on adipose tissue accumulation, an association between obesity or lipedema and lymphoedema is plausible. Chachaj et al. analysed lymphoscintigraphic alterations in lower limbs of 82 patients (51 with lipedema, 31 with obesity or overweight) and reported that both obesity and lipedema can be associated with secondary lymphoedema and additional lymphatic vessels in the affected tissue. Furthermore, their findings suggest that clinical features such as orthostatic oedema and telangiectasia in patients with lipedema and patients with obesity are highly similar [61]. Both conditions are also characterized by infiltration of immune cells, including macrophages and lymphocytes, in the affected tissue [45,55]. In a study of 258 women, Pereira de Godoy et al. showed that, although lymphoedema can occur in all patients with lipedema regardless of BMI, obesity acts as an aggravating factor, with lymphoedema prevalence increasing progressively with the body weight [62]. Because participants had no clinical signs of lymphoedema, these findings are more appropriately interpreted as evidence of subclinical lymphatic alterations or lymphatic overload than of established secondary lymphoedema. The abnormalities were predominantly minor or moderate and did not differ significantly between groups; lymphoscintigraphy therefore did not discriminate lipedema from overweight or obesity, and the cross-sectional design does not establish causal direction [61]. A cautious working model is that genetic or hormonal susceptibility may alter regional adipose-tissue behaviour; adipocyte expansion and extracellular-matrix remodelling may then increase tissue pressure and impair microvascular and lymphatic exchange. Fluid accumulation, hypoxia, and immune-cell recruitment may amplify fibrosis, pain, and further tissue expansion. Coexisting obesity may intensify several steps by increasing adipose-tissue mass, systemic inflammation, mechanical load, and lymphatic demand. This model integrates reported findings but remains hypothetical because prospective temporal and interventional evidence is lacking.

### Clinical implications and staged interdisciplinary management

#### Diagnostic confusion between lipedema and obesity affects treatment and quality of life

Although lipedema is characterized by disproportionate adipose-tissue accumulation in the extremities together with symptoms such as tension, heaviness and pain, it can remain difficult to distinguish from generalized or predominantly peripheral obesity in clinical practice. Misclassification may delay lipedema-specific care or expose patients with primary obesity to inappropriate, potentially surgical, treatment. Excess limb volume should not automatically be labelled as lipedema, but obesity assessment should likewise not rely on BMI alone. Waist circumference or waist-to-height ratio, body-composition assessment when available, adiposity-related medical complications, and functional impairment should complement BMI, particularly because disproportionate peripheral adiposity can affect BMI without defining the distribution or dysfunction of adipose tissue [1,2]. Reported onset occurs before age 30 in 70% of affected patients, whereas only 1.6% are diagnosed with lipedema before that age [19,63]. Delayed diagnosis may postpone targeted treatment, and approximately 20% of patients are first diagnosed at stage III, when physical health is more severely impaired [19]. Patients may also experience stigma when limb enlargement is attributed solely to lifestyle-related obesity. Overweight and obesity are common in referred lipedema cohorts and may aggravate symptoms, but the strength and generalizability of this association remain limited by selection bias and diagnostic heterogeneity [19–22,64].

## Weight management and multidisciplinary weight-loss interventions

Weight gain requires a sustained positive energy balance; conversely, weight loss requires a negative energy balance. A daily energy deficit of approximately 500 kcal is commonly recommended. Lifestyle modification, supplemented when indicated by formula diets, pharmacotherapy, or bariatric surgery, is a central component [65]. Accordingly, dietary modification and increased physical activity are core components of weight-management programmes [66]. Common recommendations include low-energy-density foods, reduced intake of hidden fats, regular consumption of vegetables and fruit, increased dietary fibre, and avoidance of high-calorie beverages. Dietary approaches differ primarily in macronutrient composition. Overall, macronutrient composition (e.g. low-carbohydrate or low-fat diets) has limited influence on weight loss [65]. Intermittent fasting programmes (e.g. 16:8 or 5:2) are also frequently used, although no advantage in terms of weight loss or cardiometabolic parameters has been shown in comparison to continuous energy restriction [67–69]. The inclusion of genotype information in dietary recommendations did not improve weight loss [70]. Animal studies have demonstrated changes in the gut microbiome after severe caloric restriction and subsequent weight loss [71]. Weight loss programmes like Weight Watchers or OPTIFAST can support weight reduction [72]. Self-monitoring of diet and physical activity, particularly through digital tools, can support behavioural change [73,74]. Pharmacotherapy may be added according to obesity severity, response to lifestyle treatment, comorbidities, and patient preferences. Five drugs are currently approved by the European Medicines Agency (EMA) and six by the US Food and Drug Administration (FDA) (semaglutide, tirzepatide, orlistat, phentermine/topiramate (US only), naltrexone/bupropion, and liraglutide), and can increase weight loss when combined with lifestyle treatment [75]. Reported mean weight loss is approximately 5–10%, with reductions of up to 15% reported for semaglutide and tirzepatide [65,76]. Evidence for general obesity treatment is materially stronger than the lipedema-specific evidence discussed below: several dietary and behavioural conclusions derive from randomized trials, systematic reviews, or meta-analyses, but their effects on lipedema-specific pain, disproportion, and function remain uncertain.

Bariatric surgery is an additional evidence-based option for substantial and sustained weight loss. According to the German S3 guideline cited here, surgery may be performed after conservative measures have been exhausted and may be considered earlier in patients with a BMI above 50 kg/m^2^ or with severe comorbidities that preclude postponement. The American Society for Metabolic and Bariatric Surgery recommends considering metabolic and bariatric surgery in patients with a BMI above 35 kg/m^2^, irrespective of the presence of obesity-related comorbidities. These BMI-based procedural thresholds are guideline-specific eligibility criteria and should not be confused with a comprehensive definition of obesity or adiposity-related disease. The eligibility thresholds cited here originate from guideline and consensus recommendations rather than randomized trials comparing alternative BMI or adiposity-based thresholds. Metabolic and bariatric surgery is not a lipedema-specific intervention. It should be considered only when accepted obesity indications are met, after multidisciplinary evaluation and shared decision-making, and with provision for lifelong nutritional and medical follow-up [77]. Procedure-specific contraindications and perioperative risks must be assessed independently of the lipedema diagnosis.

Bariatric procedures can be broadly classified as restrictive, reducing gastric size and capacity (e.g. sleeve gastrectomy), or as procedures that additionally induce malabsorption (e.g. Roux-en-Y gastric bypass). These approaches aim to reduce obesity-related risks and comorbidities, achieve and maintain weight loss, and improve quality of life [65].

## Effects of bariatric surgery on lipedema in patients with obesity

Because obesity may aggravate lipedema, treatment should include stabilization or reduction of body weight. Therefore, patients should receive information about the interaction between the conditions and guidance on nutrition and regular physical activity. While weight loss was long assumed not to reduce excess volume in lipedema-affected regions, Fink et al. demonstrated a significant reduction in thigh volume in patients with lipedema during weight loss after bariatric surgery, comparable to that observed in controls without lipedema [78]. Nevertheless, excess adipose tissue often persists in the extremities despite substantial weight loss. In addition, typical symptoms, including pain on pressure or touch, tension, and heaviness, frequently persist after weight loss as well [79]. Fink et al. reported a small retrospective cohort of 31 patients with lipedema and evaluated thigh volume rather than pain, function, or quality of life. The report by Bast et al. was case based. Bariatric surgery is therefore well established as an obesity treatment, whereas its lipedema-specific benefits and limitations remain supported by low-certainty observational evidence [78,79]. In the Fink cohort, adjusted thigh-volume reductions were 33.4% at the first follow-up and 30.4% at the second follow-up in the lipedema group, values similar to the comparator group. Pain, heaviness, mobility, and quality of life were not primary outcomes. These data support possible limb-volume reduction in selected patients but do not establish a lipedema-specific symptomatic effect or an expected individual response [78].

## A proposed staged interdisciplinary management framework for lipedema and obesity

**Figure 5.**
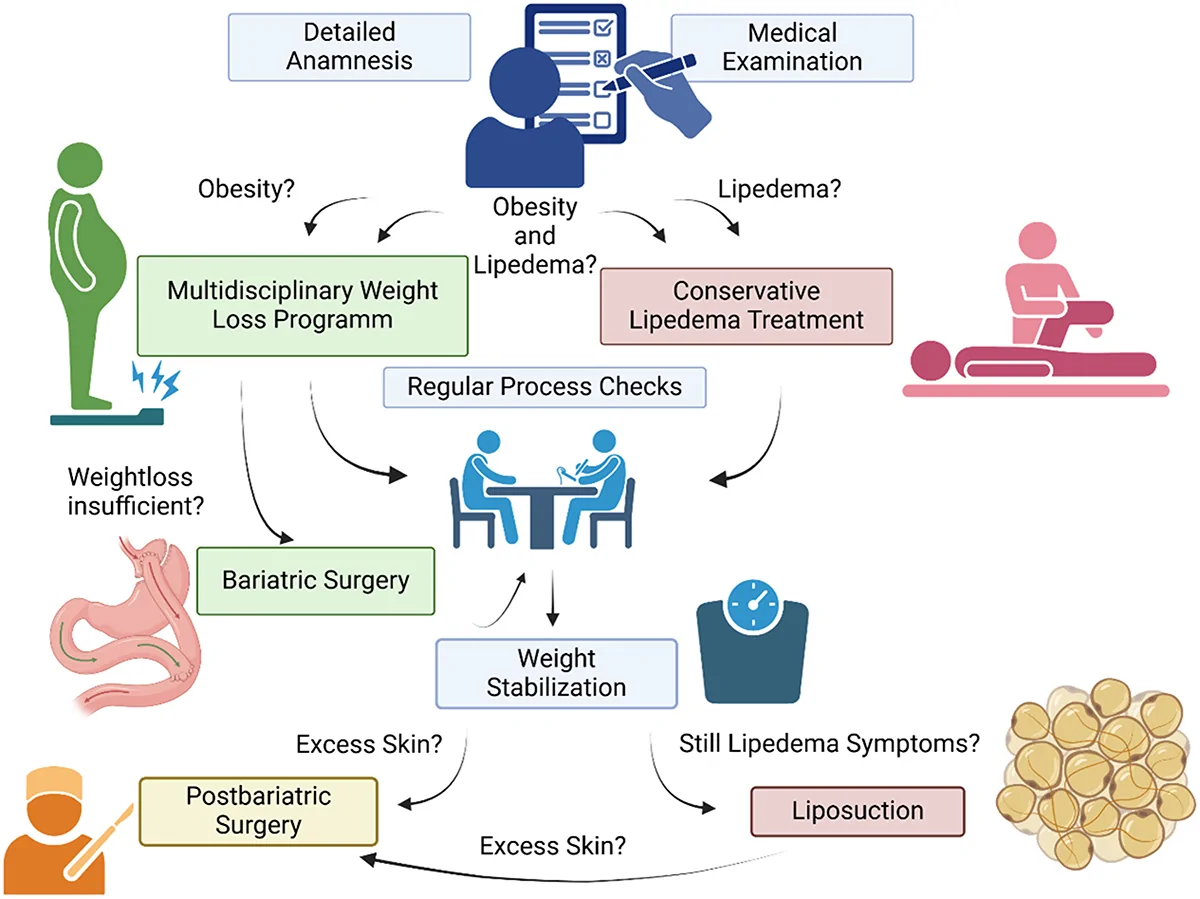
Proposed staged interdisciplinary management framework for lipedema and obesity. The framework integrates diagnostic assessment, conservative management, obesity treatment, self-management, metabolic and bariatric surgery, liposuction, post-bariatric body-contouring procedures. It is a clinical synthesis of the reviewed evidence and not a validated decision rule. Arrows indicate possible sequencing and clinical decision points, not a mandatory linear progression. Eligibility, outcome goals, follow-up domains, risks, and qualitative evidence strength are detailed in Table 3. Evidence category: expert-opinion synthesis informed by heterogeneous clinical evidence.

Management should account for the interaction among lipedema, obesity, and lymphatic dysfunction (Figure 5). The clinical history should document weight trajectories, affected regions, and the onset and progression of volume increase. Diagnostic criteria and findings that argue against lipedema, including the absence of an ankle cuff, absence of increased subcutaneous thickness, or absence of upper-to-lower-body disproportion, should be assessed systematically. Longitudinal assessment can include circumference measurements, BMI, waist circumference or waist-to-height ratio, and body-composition measures when available. Consistent with contemporary obesity frameworks, the evaluation should also document adiposity-related medical complications, psychological burden, mobility, and limitations in daily activities rather than using BMI as the sole marker of disease severity [1,2]. When lipedema is diagnosed or strongly suspected, conservative lipedema treatment should accompany multidisciplinary weight management. Pharmacotherapy or metabolic and bariatric surgery may be considered according to current guidelines and patient-specific adiposity, complications, functional burden, treatment response, and preferences; existing surgical eligibility criteria remain partly BMI-based. After weight reduction through lifestyle treatment, pharmacotherapy, or bariatric surgery, liposuction may be considered for persistent lipedema-related symptoms and disproportion. Post-bariatric body-contouring surgery may further improve quality of life and function in selected patients [80]. Expectations should be discussed explicitly: weight reduction may decrease limb volume but may not eliminate lipedema symptoms or the need for continued treatment and follow-up [22,79]. The proposed sequencing of weight management and liposuction is a clinical synthesis rather than a prospectively validated treatment algorithm. Direct liposuction evidence remains largely uncontrolled, and the evidence for post-bariatric body contouring is indirect rather than lipedema-specific. The pathway should be interpreted as a set of parallel and conditional options rather than a mandatory progression through every intervention. Diagnostic confidence, dominant symptoms, adiposity-related disease, functional limitation, prior response, procedural risk, and the patient’s priorities determine eligibility and sequencing. Conservative care, psychosocial support, and obesity treatment may proceed concurrently; bariatric surgery, liposuction, and post-bariatric body contouring are optional interventions with distinct indications. The decision points, intended outcomes, risks, follow-up domains, and qualitative evidence ratings are summarized in Table 3.

**Table 3.** Clinically oriented framework for staged and parallel management of lipedema and coexisting obesity.

B	Rationale and expected outcomes	Risks, limitations, and evidence	Follow-up and research needs
**Diagnosis – Target**: All patients with suspected lipedema, disproportionate extremity adiposity, pain, oedema, or diagnostic uncertainty.	**Rationale**: Differentiate lipedema from other causes of adipose-tissue or limb enlargement and identify coexistence. **Goals**: A defensible diagnosis, a multidomain baseline, avoidance of inappropriate treatment, and identification of obesity or lymphoedema requiring parallel care.	**Risks/limits**: No validated biomarker or single confirmatory test; heterogeneous criteria and coexistence increase misclassification risk. **Evidence**: Consensus-based clinical guidance; limited diagnostic-accuracy evidence.	**Follow-up**: Reassess when phenotype, weight, pain, oedema, or function changes; record pain, function, quality of life, volume, weight trajectory, and comorbidities. **Research**: Validated diagnostic criteria, prospective accuracy studies, and population-based cohorts.
**Conservative therapy – Target**: Symptomatic patients with confirmed or probable lipedema; oedema treatment should be tailored to the cause and clinical findings.	**Rationale**: Reduce symptom burden, support mobility, and strengthen self-management using individually selected compression, exercise, skin care, and related measures. **Goals**: Possible improvement in pain, heaviness, function, and quality of life; volume effects are variable and no reliable pooled lipedema-specific effect size is established.	**Risks/limits**: Treatment burden, garment fit, skin problems, cost, and adherence; modality-specific contraindications must be assessed. Manual lymphatic drainage does not remove adipose tissue. **Evidence**: Low direct comparative lipedema evidence; supported mainly by consensus and observational data.	**Follow-up**: Use the same pain, function, quality-of-life, treatment-burden, and volume measures over time; modify treatment for nonresponse or adverse effects. **Research**: Adequately powered trials, standardized protocols, core outcomes, and durability.
**Weight management – Target**: Patients with overweight, obesity, weight gain, adiposity-related complications, or functional impairment; assess eating behaviour and psychological context.	**Rationale**: Treat coexisting excess adiposity and adiposity-related dysfunction that may worsen mobility, metabolic risk, lymphatic load, or limb enlargement. **Goals**: Improved weight trajectory, waist measures, metabolic health, mobility, and potentially limb volume; lipedema-specific pain or disproportion may persist.	**Risks/limits**: Weight cycling, stigma, loss of lean mass, nutritional inadequacy, or worsening disordered eating if poorly individualized. Lipedema is not explained by lifestyle alone. **Evidence**: Strong evidence for general obesity care; low certainty for lipedema-specific symptom effects.	**Follow-up**: Track weight, waist or waist-to-height ratio, body composition when available, metabolic outcomes, mobility, pain, and patient-defined goals. **Research**: Predictors of limb and symptom response, long-term stability, and lipedema-specific outcomes.
**Pharmacotherapy – Target**: Standard obesity indications and agent-specific eligibility; lipedema alone is not an indication.	**Rationale**: Adjunctive treatment of obesity when lifestyle measures alone are insufficient or complications warrant escalation. **Goals**: Drug-dependent weight and metabolic benefits; a lipedema-specific effect is unproven.	**Risks/limits**: Agent-specific contraindications, adverse effects, access and cost; weight regain after discontinuation may occur. **Evidence**: Moderate to high for approved agents in general obesity populations; very low or absent for lipedema-specific outcomes.	**Follow-up**: Assess weight and metabolic response, adverse effects, adherence, pain, function, and whether the drug remains indicated; stop or switch according to obesity guidance. **Research**: Prospective lipedema subgroup studies with symptoms, function, and limb outcomes.
**Metabolic and bariatric surgery – Target**: Only patients meeting accepted obesity-surgery indications after multidisciplinary evaluation and shared decision-making; lipedema alone is not an indication.	**Rationale**: Provide substantial and durable obesity treatment and reduce obesity-related disease; may reduce thigh volume when lipedema and obesity coexist. **Goals**: Established general obesity benefits. In one small retrospective lipedema cohort, adjusted thigh-volume reduction was 33.4% at first and 30.4% at second follow-up; symptom benefit remains uncertain.	**Risks/limits**: Operative and anaesthetic complications, nutritional deficiencies, reoperation, weight regain, and lifelong surveillance. Procedure-specific contraindications and ability to engage in follow-up must be assessed. **Evidence**: High for general obesity outcomes; very low for lipedema-specific volume and symptom outcomes.	**Follow-up**: Lifelong nutritional and medical follow-up; record weight, complications, limb volume, pain, heaviness, mobility, quality of life, and residual tissue after weight stabilization. **Research**: Controlled lipedema studies with patient-reported outcomes, longer follow-up, and predictors of response.
**Liposuction – Target**: Confirmed lipedema with documented treatment-resistant pain and/or relevant functional, dermatological, or orthopaedic complications. Selection should not rely on morphological stage alone; coexisting obesity and oedema require prior or parallel management.	**Rationale**: Reduce painful or mechanically burdensome subcutaneous adipose tissue when clinically relevant symptoms persist despite appropriate conservative management. **Goals**: Observational cohorts report improvement in pain, heaviness, bruising, mobility, quality of life, and conservative-treatment burden, but complete symptom resolution is not assured and a reliable individual effect size is unavailable.	**Risks/limits**: Bleeding, infection, thromboembolism, seroma, contour problems, anaesthetic risk, possible lymphatic injury, multiple procedures, and persistent symptoms. BMI > 40 kg/m^2^ plus waist-to-height ratio > 0.55 is a critical indication constellation in the S2k guideline, not an absolute contraindication. **Evidence**: Low certainty: predominantly uncontrolled observational studies; meta-analysis inherits primary-study bias.	**Follow-up**: Monitor complications, pain, heaviness, mobility, quality of life or PBI-L, limb measures, compression dependence, and weight trajectory; reassess goals before additional procedures. **Research**: Controlled comparative trials, standardized techniques and outcomes, responder profiles, harms, and long-term durability.
**Post-bariatric body contouring – Target**: Selected patients after weight stabilization and nutritional optimization; not a routine lipedema treatment.	**Rationale**: Address excess skin or residual tissue after major weight loss when it impairs hygiene, function, mobility, or quality of life. **Goals**: Possible functional and quality-of-life improvement; the lipedema-specific contribution is uncertain.	**Risks/limits**: Wound complications, seroma, infection, scarring, anaesthetic and lymphatic risks, and need for multiple operations. **Evidence**: Moderate to low for general post-weight-loss populations; very low for lipedema-specific outcomes.	**Follow-up**: Assess wound healing, nutrition, function, quality of life, lymphatic symptoms, weight stability, and patient-defined benefit. **Research**: Prospective lipedema cohorts, optimal timing, procedure selection, and long-term outcomes.
**Psychosocial care – Target**: Screen all patients; offer targeted support when distress, stigma, disordered eating, or impaired self-management is present.	**Rationale**: Address distress, stigma, chronic-pain coping, depression or anxiety, disordered eating, body image, and barriers to self-management. **Goals**: Improved coping, goal setting, participation, adherence, and quality of life; psychosocial care does not imply that lipedema is psychogenic.	**Risks/limits**: Limited access and risk of invalidation or additional stigma if care is framed poorly. **Evidence**: Moderate indirect evidence from chronic-disease and obesity care; limited lipedema-specific trials.	**Follow-up**: Use validated distress and quality-of-life measures, assess social participation and treatment burden, and revisit goals across transitions in care. **Research**: Lipedema-specific interventions, stigma reduction, and patient-defined outcome thresholds.
**Long-term follow-up – Target**: All patients, especially after treatment escalation or surgery.	**Rationale**: Identify benefit, nonresponse, complications, weight regain, evolving lymphoedema, and changing patient priorities. **Goals**: Earlier detection of harm or nonresponse, maintenance of benefit, and iterative adjustment of goals and treatment intensity.	**Risks/limits**: No agreed follow-up interval or core outcome set; measurement variability and treatment burden. **Evidence**: Expert-opinion synthesis supported by general chronic-disease principles.	**Follow-up**: Use the same baseline and serial measures; review pain, heaviness, function, quality of life, limb measures, weight, comorbidities, treatment dependence, adverse events, and goals in shared decisions. **Research**: Core outcome set, minimal clinically important differences, optimal intervals, and long-term comparative pathways.

Treatment success should be defined prospectively using domain-specific outcomes rather than weight or limb volume alone. Relevant outcomes include lipedema-related pain, heaviness, bruising, mobility, daily functioning, quality of life, limb volume and disproportion, adiposity-related complications, adverse events, and the continuing need for conservative treatment. Shared decision-making should address the specific target of each intervention, alternatives, uncertainty, treatment burden, and the possibility that anatomical and symptomatic outcomes may diverge. Baseline and follow-up assessments should use the same predefined domains. Pain can be recorded with a 0–10 visual analogue or numerical rating scale; patient-relevant benefit can be assessed with the validated Patient Benefit Index for Lymphoedema and Lipedema (PBI-L), while generic instruments such as the SF-36 or EQ-5D permit broader quality-of-life assessment [17,81]. Mobility should be documented with a reproducible functional measure appropriate to the patient, and anatomical change with standardized circumference or volume measurements. Weight trajectory, waist circumference or waist-to-height ratio, body composition where available, obesity-related complications, compression or manual-therapy dependence, adverse events, psychosocial distress, and stigma should be followed longitudinally. Because available measurement protocols are heterogeneous and no single physical measure is an established diagnostic or outcome gold standard, the method and anatomical landmarks should be reported consistently [82]. From the patient perspective, successful treatment is the achievement of prespecified personally relevant goals, such as meaningful reduction in pain or heaviness, improved mobility or daily functioning, better quality of life or metabolic health, or reduced treatment burden, without unacceptable adverse effects. Normalization of limb shape or body weight is neither necessary nor sufficient to define success.

## Limitations of the review approach

This narrative review has methodological limitations. PubMed was the principal bibliographic database, and the supplementary Embase and Cochrane Library searches were focused rather than designed as an exhaustive systematic search. Publication selection involved author judgement and may therefore be affected by selection bias, while relevant studies indexed exclusively elsewhere may have been missed. No formal risk-of-bias assessment or quantitative synthesis was performed. The manuscript should consequently be interpreted as a clinically oriented narrative synthesis, not as a systematic or scoping review and not as a comparative-effectiveness assessment.

## Conclusion

Current evidence supports a clinically important interaction between lipedema and obesity, but it does not establish a single causal pathway. A plausible model links regional adipose-tissue susceptibility and expansion with extracellular-matrix remodelling, vascular permeability, lymphatic stress, hypoxia, and local immune-cell recruitment; coexisting obesity may amplify several of these processes. Most molecular and histological findings remain observational, and proposed initiating factors and amplification mechanisms should therefore be distinguished from established clinical features.

Diagnostic assessment should identify lipedema on clinical grounds while evaluating obesity as an adiposity-based chronic disease rather than a BMI category alone. BMI can support screening and treatment eligibility, but adipose-tissue distribution, waist measures or body composition, medical complications, functional limitations, and psychological burden are needed to characterize the individual patient. A staged interdisciplinary approach may combine conservative lipedema care, structured obesity treatment, self-management, pharmacotherapy, bariatric surgery, liposuction, and post-bariatric body-contouring procedures according to the dominant symptoms, risks, and patient goals. Benefit should be judged using defined outcomes such as pain, function, quality of life, limb disproportion, metabolic health, treatment burden, and adverse events. No single anatomical or procedural endpoint should be used as a universal definition of treatment success.

The evidence base remains limited by heterogeneous diagnostic criteria, clinic-based recruitment, small and cross-sectional mechanistic studies, and few controlled comparisons of treatment sequences. Future research should prioritize standardized diagnostic criteria, population-based epidemiology, prospective longitudinal studies that establish temporal relationships, and comparative trials with predefined clinical and patient-reported outcomes. Until such evidence is available, interdisciplinary management should be presented as a reasoned and individualized framework rather than as a definitively successful treatment algorithm.

## Data Availability

Data sharing is not applicable as because no new data were generated or analysed in this study.
